# Some novel intron positions in conserved Drosophila genes are caused by intron sliding or tandem duplication

**DOI:** 10.1186/1471-2148-10-156

**Published:** 2010-05-26

**Authors:** Jörg Lehmann, Carina Eisenhardt, Peter F Stadler, Veiko Krauss

**Affiliations:** 1 Bioinformatics Group, Department of Computer Science, and Interdisciplinary Center for Bioinformatics, University of Leipzig, Härtelstraße 16-18, 04107 Leipzig, Germany; 2 Genetics Group, Department of Biology II, University of Leipzig, Johannisallee 21-23, 04103 Leipzig, Germany; 3 Max Planck Institute for Mathematics in the Sciences, Inselstraße 22, 04103 Leipzig, Germany; 4 RNomics Group, Fraunhofer Institute for Cell Therapy and Immunology, Perlickstraße 1, 04103 Leipzig, Germany; 5 Institute for Theoretical Chemistry, University of Vienna, Währinger Straße 17, 1090 Wien, Austria; 6 Santa Fe Institute, 1399 Hyde Park Rd., Santa Fe, NM 87501, USA

## Abstract

**Background:**

Positions of spliceosomal introns are often conserved between remotely related genes. Introns that reside in non-conserved positions are either novel or remnants of frequent losses of introns in some evolutionary lineages. A recent gain of such introns is difficult to prove. However, introns verified as novel are needed to evaluate contemporary processes of intron gain.

**Results:**

We identified 25 unambiguous cases of novel intron positions in 31 Drosophila genes that exhibit near intron pairs (NIPs). Here, a NIP consists of an ancient and a novel intron position that are separated by less than 32 nt. Within a single gene, such closely-spaced introns are very unlikely to have coexisted. In most cases, therefore, the ancient intron position must have disappeared in favour of the novel one. A survey for NIPs among 12 Drosophila genomes identifies intron sliding (migration) as one of the more frequent causes of novel intron positions. Other novel introns seem to have been gained by regional tandem duplications of coding sequences containing a proto-splice site.

**Conclusions:**

Recent intron gains sometimes appear to have arisen by duplication of exonic sequences and subsequent intronization of one of the copies. Intron migration and exon duplication together may account for a significant amount of novel intron positions in conserved coding sequences.

## Background

**Figure 1:**
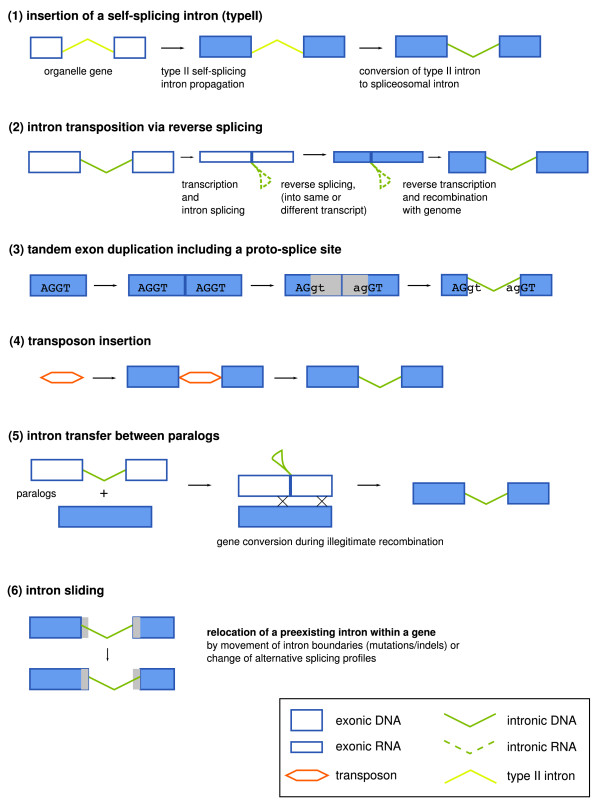
**Overview on mechanisms proposed for the emergence of novel intron positions within conserved ORFs**. It is commonly assumed that the first mechanism was responsible for the emergence of spliceosomal introns in ancient eukaryotes. More recent intron gains or intron position changes may be caused by the other mechanisms shown.

Comparative studies of spliceosomal intron densities have suggested relatively high rates of intron gain during eukaryote evolution [1,2]. The establishment of introns within fast-evolving genes appears to be an infrequent, but common process involving, for example, intronization of exonic sequences [3-5]. However, recent gains of introns inside of conserved coding sequences (CDS), often equated with the usage of novel intron positions, appear to be a rare and poorly understood phenomenon [6]. At least six mechanisms (see Figure 1 for a schematic overview) have been proposed to explain novel intron positions within conserved open reading frames (ORFs): (1) insertion of a self-splicing type II intron via reverse splicing [7]; (2) insertion of a spliceosomal intron via reverse splicing into a new position [8]; (3) partial tandem duplication of an exon including a cryptic AG/GY splice motif [7]; (4) insertion of a transposable element [9]; (5) gene conversion from an intron-containing site into a previously intron-less paralogous site [10]; and (6) intron sliding [11]. Only the last three pathways are supported by undisputed, albeit anecdotal, evidence [10,12,13]. Recently, a study on *Daphnia *populations [14] suggested another intron gain mechanism: The repair of DNA double-strand breaks using small segmental insertions.

The analysis of near intron pairs [15] allows a systematic investigation of intron gain mechanisms. A near intron pair (NIP) consists of two intron positions that exist in orthologous genes at nearby locations. Exon sizes smaller than about 50 nt are relatively rare [16] and in general functionally detrimental [17]. Thus, such nearby introns typically exclude each other within a single gene. Accordingly, one of these introns must be evolutionarily younger and should define a monophyletic group. By using NIPs to determine the time at which the younger introns were gained, it can be avoided to erroneously identify those introns as novel that have been lost independently in multiple lineages.

Here we use the relatively recently diverged genomes of 12 Drosophila species [18] to identify recent intron gain events in a comparative analysis of gene structures and evaluate possible mechanisms of their origin. In contrast to previous attempts to identify intron loss and gain in Drosophila [19,20], we chose the NIP approach instead of Dollo parsimony to restrict our analysis to introns for which the evidence of intron gain is more unambiguous. We could identify 31 NIPs within Drosophila.

Their distribution supports the known species phylogeny. Both introns of a NIP were evaluated for sequence similarity to introns, neighboring exons and transposable elements and screened for repetitiveness and potentially meaningful secondary structures, using the ancient, plesiomorphic intron of the NIP as control. In addition, we looked for cryptic splice signals in the adjacent exonic and intronic sequences. There is evidence for intron sliding in 9 of the 31 Drosophila NIPs, while 5 other cases probably arose by tandem duplications within the ORF. In contrast, we found no evidence for any intron insertion mechanism based on alien sequences in our data set.

## Results and Discussion

### Compilation and characterization of the NIP data set

We started with 12386 sets of orthologous protein-coding genes of Drosophila species (Methods). After an automatic NIP extraction alignment procedure, we obtained 122 NIP regions containing two or more near introns (NIP distance < 50 nt). These were manually inspected for splice site, alignment and conservation validity, resulting in 40 regions comprising 41 NIPs. Based on the frequency of short exons and NIP distances within Drosophila (data not shown), we decided to decrease the maximal allowed intron distance to 31 nt, which resulted in 35 alignment regions comprising 36 NIPs (Additional files 1 and 2).

Since there was an alternative site consistent with the old (plesiomorphic) intron position that could not be clearly excluded by splice site sequence analysis, we evaluated seven NIP candidates experimentally. As no EST data supported these NIPs, we performed RT-PCR or genomic PCR experiments in some crucial species (Additional file 3). Our analysis confirmed 2 of the investigated NIPs (FBgn0015572, FBgn0046689). The remaining 5 cases were reducible to one intron position. Three of these cases were based on sequence errors that could be corrected by genomic PCR or RT-PCR (FBgn0036324, FBgn0027055, FBgn0046689). One NIP candidate represented a frame shift mutation that led to an incorrectly annotated splice site (FBgn0038858). In the last case (FBgn0082831), intron sliding has happened only at the 3' splice site.

### Age of NIPs

Not all of the remaining 31 NIPs contain introns that necessarily have been gained during the evolution of the genus Drosophila. Using the intron distributions among Drosophila and the arthropod outgroup species *Glossina morsitans, Aedes aegypti, Anopheles gambiae, Culex pipiens, Bombyx mori, Tribolium castaneum, Apis mellifera, Nasonia vitripennis, Acyrthosiphon pisum, Pediculus humanus, Daphnia pulex *and *Ixodes scapularis*, we discovered that at least 25 intron positions of the 31 NIPs have arisen during Drosophila radiation. 17 NIPs contain exactly one novel intron position, whereas 4 other NIPs consist of two different novel intron positions, which are supported by other nearby introns in the outgroup species. The phase distribution of these 25 novel introns is similar to the average intron phase distribution in Drosophila [19]: 13 of these introns are in phase 0 (52%), and 6 in phase 1 and phase 2, respectively (24%). For the 10 remaining NIPs, the relative age of intron positions could not be determined either due to a lack of sufficient local sequence similarity to orthologous gene structures outside of Drosophila or due to a lack of nearby introns in outgroup sequences.

### NIPs are suitable phylogenetic markers for Drosophila species

**Figure 2:**
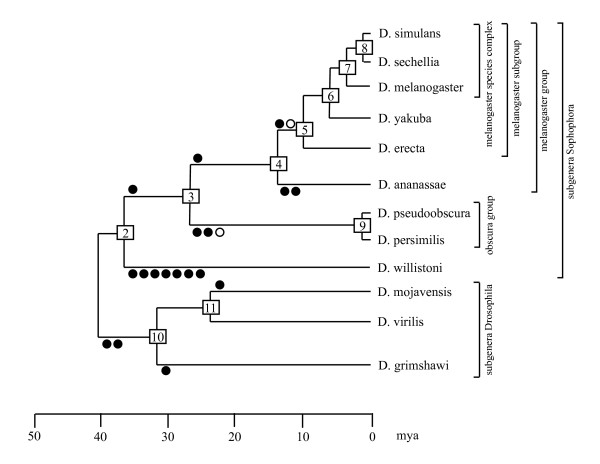
**Apomorphic introns, mapped onto the Drosophila tree**. Changed intron positions that could unambiguously be mapped onto a specific branch are denoted by circles. Filled circles indicate intron changes that occurred only once. Two empty circles indicate two independent slidings of a single ancient intron into the same derived position. The tree is scaled according to the DroSpeGe database [21].

NIPs were primarily introduced as reliable phylogenetic markers for insect evolution [15]. The well-known phylogeny of Drosophila [18] opens the possibility to verify the suitability of NIPs for phylogenetic analyses of recent radiations (Figure 2). Phylogenetically informative NIPs within Drosophila were identified using the aforementioned arthropod outgroup species. Together, 7 synapomorphic (shared derived) and 11 autapomorphic (species-specific) NIP characters appeared during the evolution of the genus Drosophila. The subgenus *Drosophila*, the species groups *obscura *and *melanogaster*, the sister relationship between the *melanogaster *and the *obscura *species groups and as well as the *melanogaster *species subgroup are supported by at least one synapomorphically distributed NIP (Figure 2). For 4 remaining internal nodes (subtrees) and 8 remaining external nodes (species) of the tree no supporting NIP evidence was found.

Out of 31 NIPs, only one character distribution (FBgn0003607, intron positions 117-1 and 118-1) contradicts the established tree of the 12 Drosophila species. This NIP is clearly associated with a sliding event (see below). The local nature of sliding makes independent changes of intron position with identical results much more likely than any of the other mechanisms, which require independent targeting of the same genomic position. We conclude that NIPs could be reliable phylogenetic markers also for recent radiations. However, short branch lengths, as observed between Drosophila species, could critically limit the number of available characters. Intron sliding accounts for a low but detectable level of homoplasy in this type of character.

### Intron migration into novel positions

**Figure 3:**
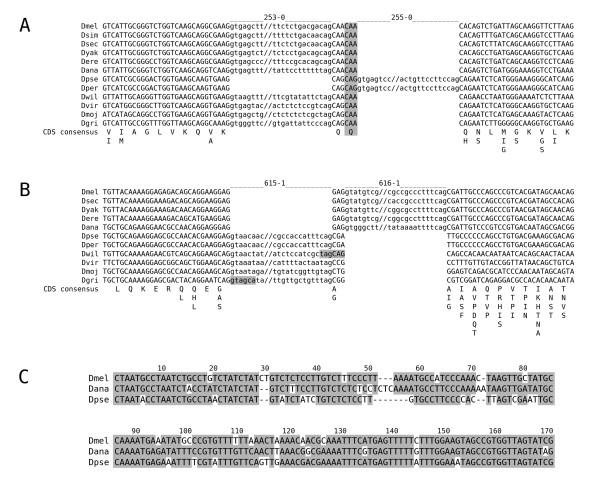
**Examples for intron sliding**. Note that all Drosophila species have an intron in these regions and that position changes may have taken place in two steps as the frame would have been maintained in between. (A) NIP region of the gcm2 gene (FBgn0019809). Intron sequences are shown in lower case. The amino acid sequence consensus of the CDS is given beneath the exon sequences. The second conserved glutamine codon between both intron positions has probably supported the shift in a common ancestor of *D. pseudoobscura *and *D. persimilis*. This conserved, potential 3' splice site is highlighted in grey. (B) NIP of the CG8516 gene (FBgn0037757). Intron sequences are shown in lower case. The amino acid sequence consensus of the CDS is given beneath the exon sequences. GYRGYR/NAGNAG sites corresponding to the intron shift are highlighted in grey. (C) Conserved part of the intron sequences 616-1 (*D. melanogaster, D. ananassae*) and 615-1 (*D. pseudoobscura*) of the CG8516 NIP. Each intron is larger than 300 nt.

**Figure 4:**
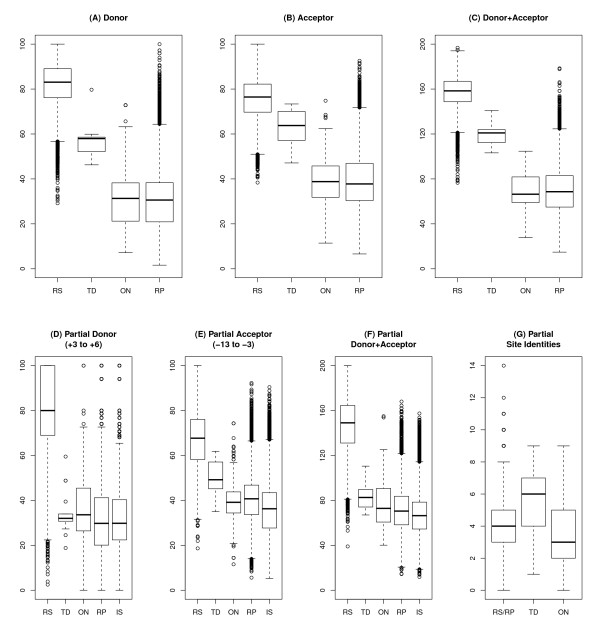
**Scores of proto-splice sites**. The distributions of splice site scores are shown separately for donor, acceptor, and the sum of donor and acceptor sites, displaying different sets of sites: (RS) 16766 reference splice sites of *D. melanogaster*, (TD) the 18 proto-splice sites from 7 apomorphic NIP introns potentially arised by tandem duplications, (ON) the 236 proto-splice sites from all other NIP introns, (RP) 16506 potential proto-splice sites corresponding to the reference introns, and (IS) 52626 exonic AGGY motif surroundings which are located more than 31 nt apart from exon boundaries. **(A-C) **Results that refer to complete splice consensus sites. TD proto-splice sites are potentially functional splice sites as (1) they are within the range of functional splice site scores of the reference set (RS), and (2) they reside within the top range of proto-splice site scores of the intron reference set (RP). **(D-F) **Scores determined as in (A-C) that refer to a partial splice consensus excluding the motif AG/GY. **(G) **Identical nts between the donor and acceptor sites of each intron excluding the motif AG/GY. 15 nts (-13 to -3 and +3 to +6 in relation to start and end of each intron, respectively) were compared between both sites. Given no similarity between both sites, 3.75 identical nt positions would be expected at random.

Next, we evaluated possible evolutionary mechanisms that had produced introns at novel positions. For all the introns of the 31 NIPs, we could not find any relevant sequence conservation to other introns, to transposons, or to exons (data not shown). To control whether some of the intron position changes resulted from intron sliding, we evaluated all introns of the NIPs concerning properties that would be expected if a coordinated migration of both splice sites occurred in relation to the former intron and the CDS. These properties are: (1) There is no intermediate, intron-less state in the tree; (2) NIP distance is a multiple of 3 allowing a stepwise intron shift; (3) cryptic splice sites occur at NIP distances and are supported by amino acid conservation (Figure 3A); (4) one-sided shifts or GYRGYR/NAGNAG splice sites in some species (Figure 3B); and (5) significant sequence similarity (Materials and Methods) between introns of both positions (Figure 3C). The last criterion is sufficient on its own to support intron sliding and was found in 3 cases (Table 1). Here, we observed that the intron sequence had stayed in place, but that the splice sites seem to have been migrated.

**Table 1:** Properties and origins of NIPs.

nt	FBgn	apomorphic introns	Properties typical for intron sliding					Properties typical for tandem duplication	
			**No intermediate, intron-less state in the tree**	**Multiple-of-three NIP distances that made a stepwise intron shift possible**	**Cryptic splice sites, occurring in NIP distance and supported by amino acid conservation**	**One-sided shifts or GYRGYR/NAGNAGs in some species**	**Significant sequence similarity between introns of both positions**	**Proto-splice site (AG/GY)**	**plesiomorphic introns not identifiable**

**probably migrated intron positions**									

3	3607	118-1	x	x		x			

3	32261	55-0	x	x		x			

3	37757	616-1	x	x		x	x		

6	19809	255-0	x	x		x			

6	35879	105-1	x	x	x		x		

9	32821	1200-0	x	x	x				

9	34221		x	x			x		x

12	33734	419-0, 423-0	x	x		x			

15	1124	124-0	x	x	x				

**introns probably gained by tandem (exon) duplication**									

10	30661							x	

21	29747		x	x				2x	x

30	38300		x	x				2x	x

30	50101	251-0	x	x				x	

31	2526							x	x

**NIPs of unknown origin**									

2	31216	1927-1, 1928-0	x						x

2	36142	456-0, 456-2	x		x				

3	15572	4-2	x	x					

3	32087	19-1	x	x					

3	32504			x					x

3	52081		x	x					x

5	46689	16-2	x		x				

6	31395		x	x					x

9	38302	65-2	x	x					

14	35965	44-0	x						

16	33686	53-2, 59-0	x						x

21	30055		x	x					x

22	32517	221-0	x						

23	33247	45-2	x						

24	34793	29-0	x	x					

27	1185			x					x

28	31773	164-1	x						

∑	**31**	**25**	**27**	**21**	**5**	**5**	**3**	**7**	**11**

"nt" means NIP distance in nucleotides. Note that each of the 9 NIPs resulting supposedly from intron sliding shows at least three of the expected properties and/or a significant sequence similarity between introns of both positions (Materials and Methods). Introns gained by tandem duplication show a proto-splice site. In addition, a plesiomorphic intron is not identifiable because both introns probably have been gained by the same duplication.

In another 6 NIPs, at least three of the other four criteria are fulfilled. While the first two conditions are also consistent with mechanisms of intron gain (see below), cryptic splice sites at the corresponding distance and one-sided shifts of intron borders in some species are specific requirements for intron sliding (Figures 3A and 3B). Thus, the fulfillment of at least three criteria argues for sliding also in these cases. It should be noted that only one case of sliding (FBgn0034221) has occurred within about 6 million years of evolution (between *D. yakuba *and *D. melanogaster*) [21] and was, therefore, expected to show sequence conservation between both introns of the NIP. All other putative sliding events have occurred between Drosophila lineages that diverged at least 14 million years ago (*D. ananassae *and *D. melanogaster*). No footprint of internal sequence conservation across the two intron positions has been retained in any of these cases.

In summary, 9 out of 31 NIPs most likely originated by intron sliding. This is surprising since earlier studies concluded that intron sliding is a very rare event [13]. On the other hand, frequent intron sliding can be expected as a consequence of the high abundance of tandem splice sites (GYRGYR/NAGNAG) in eukaryotic genes [22].

### Detection of strong proto-splice sites in a subset of novel introns

Donor and acceptor site consensus sequences of spliceosomal splicing also include exonic parts. At the donor (5') splice site, the last two exonic nucleotides (typically 5'-AG-3') could bind to the U1 snRNA [23] and the corresponding nucleotide positions are essential in some human splice sites [24,25]. In contrast, the acceptor (3') splice site appears to be functionally independent from exonic parts of the consensus [26]. Surprisingly, there are nucleotide preferences nevertheless, typically 5'-RT-3' [27]. Sverdlov et al. [28] reported that younger introns exhibit a stronger signal in the exonic part of both splice consensus parts than older ones, while the intronic part of the splice signal is stronger for older introns than for younger ones. Both observations are to be expected if novel introns sometimes originate through tandem duplications within coding exons (exon duplication). Following such an event, a duplicated proto-splice site (consisting of a potential intronic splice acceptor immediately followed by a potential intronic splice donor) could turn the sequence between both sites into an intron (Figure 1) [29]. In Drosophila, the role of the branch site and of additional enhancer and silencer sequences is very limited [30] and typically does not interfere with an intron definition by donor and acceptor site alone. Thus, an intron could immediately emerge by tandem duplication of a proto-splice site and will have the size of the duplication.

Concomitantly, such a duplication will allow differential splice patterns (intron retentions) as a temporary or permanent alternative to the establishment of a constitutive intron if the duplication size is a multiple of three. Alternatively, unspliced mRNA variants may be degraded by nonsense-mediated decay (NMD) [31]. Thus, NMD may often serve as a backup for weak splicing of such novel introns as recently suggested by Farlow et al. [20]. The mature mRNA will remain qualitatively unchanged during such a process. Later on, the splice signals could be improved by selection, whereas the other parts of the novel intronic sequence are free to change by insertions, deletions and substitutions.

Thus, we searched for coincidences of introns and proto-splice sites supported by amino acid conservation. We found 9 introns in 6 NIPs that are surrounded by an AG/GY proto-splice site. For 7 of these introns (from 5 NIPs; referred to later on as TD introns), the proto-splice sites are conserved also in Drosophila species having no intron there, and should thus already have been present at the time of intron origin. The potential functionality of these sites both as donor and acceptor sites is supported by their splice site scores (calculated from the exonic sites -13 to +6, Materials and Methods) that reside within the variability of functional splice sites of *D. melanogaster*, but well above the splice site scores of typical exonic sequences surrounding Drosophila introns (Figure 4A-C, Table 2). For two of these 7 TD introns, regional repetitiveness enhances the probability of duplications in the CDS (FBgn0002526 and FBgn0050101). In all 18 individual sequences of TD introns we found a premature termination codon, suggesting that NMD might have played a role during their evolution.

**Table 2:** Intron gain by tandem duplication as suggested by proto-splice sites.

FBgn	Intron	Proto-splice site consensus of the surrounding CDS (all Drosophila species in alignment)	Percentile of the smallest score per position within the reference set RS (RP)		
					
		Splice site consensus of introns			
0002526	1460-0	CAGGTSATT//YGGATTCCATCAGG	2.99 (96.61)	//	7.52 (92.18)
		
		CAGgtaagw//cattgtccaccagG	75.49	//	18.99

0029747	200-0	CAGGTHCTH//YAARMGWKTRCAGG	0.38 (91.34)	//	9.77 (93.46)
		
		CAGgtaskk//bshsuymywdyagG	1.23	//	11.28
	
	207-0	CAGGCACGC//CAAGTCAYTGCAGG	0.48 (92.31)	//	21.59 (96.80)
		
		CAGgtrrgy//tktcssmtkgcagG	29.43	//	30.97

0030661	211-0	GAGGTTATC//CCGAAATTTTGAGG	1.72 (95.60)	//	0.25 (74.97)
		
		GAGgtgaga//agtgcactttcagG	56.04	//	38.86

0038300	44-0	CAGGCGCTT//TCAATGCCTGCAGG	0.23 (88.73)	//	36.08 (98.49)
		
		CAGgtaagc//cgtttatttttagG	72.27	//	69.96
	
	54-0	AAGGTGGAG//RCCGCCTTCMAAGG	2.22 (96.17)	//	2.47 (86.57)
		
		AAGgtaagw//hbyymykukyyagG	80.74	//	8.28

0050101	251-0	AAGGTGCCC//CGTCCATATCAAGG	0.90 (94.17)	//	3.97 (89.17)
		
		AAGgtaaga//gttaatcatctagG	80.74	//	17.36

For details of the reference data set and score generation see the Methods section. Percentile values in brackets are with respect to the RP reference set. Consensus nts: B = not A, D = not C, H = not G, K = G or T, M = A or C, R = A or G, S = C or G, U = not T, W = A or T, Y = C or T.

### Identified proto-splice sites support tandem duplication rather than reverse splicing

The concept of the proto-splice site was proposed more than 20 years ago [32]. Despite the simplicity of the tandem duplication mechanism (also known as exon duplication), intron gain was seldom explained in this way [19,33,34, but see 35]. Instead, proto-splice sites were introduced as preferential insertion sites for reverse-spliced introns [36]. This pathway requires four successive, rate-limiting steps, namely (1) germ line transcription of the target gene; (2) reverse splicing of an - occasionally retained - intron lariat into a novel site of the target mRNA; (3) reverse transcription of this now intron-containing mRNA; and (4) homologous recombination of this cDNA with the target gene. To our knowledge, this reverse-splicing pathway has never been shown to have produced a spliceosomal intron.

In contrast, tandem duplications frequently occur in natural populations. Emerson et al. [37] compared 15 natural isofemale lines of *D. melanogaster *and detected 1901 duplications, mostly in tandem, with a median size of 367 nt in at least one line. Irrespective of the strong evidence for purifying selection against this type of mutations, 624 of these duplications included some exonic sequences, but not a whole gene. Given this significant amount of function-challenging mutations, the gain of an intron may compensate for a duplication simply if a proto-splice site is included.

In order to further support the hypothesis that the 7 TD introns arose by tandem duplication rather than by reverse splicing, we evaluated their proto-splice sites. If splice sites had emerged directly by duplication of proto-splice sites, (1) proto-splice and splice sites should be similar to each other and (2) both should be functional splice sites. If intronic splice sites stemmed from a reverse-spliced intron, proto-splice sites would not need to be similar to splice sites but should have provided a preferential binding site for the spliceosome that has inserted the intron. The sequence specificity of spliceosome-binding during forward splicing is mediated by the binding of the U1 snRNP to the donor site [23]. However, the initial interactions of a spliceosome capable of reverse splicing are unknown. Thus, alternatively, the U5 snRNP complex maybe binds first because this complex interacts during the second splicing reaction with both exon ends to ligate them [38]. For U5 snRNP, known sequence preferences are weak and not consistent with proto-splice sites [39]. Other sequence-specific binding components of the spliceosome such as the U2, U4 and U6 snRNPs exclusively interact with the intron. Reverse splicing, therefore, might have produced proto-splice sites that remember donor sites (nts -2 to +6: AG/GYRAGT) or no proto-splice sites at all. Specifically, the acceptor site consensus 5' of AG would not necessarily be included in such a proto-splice site.

**Figure 5:**
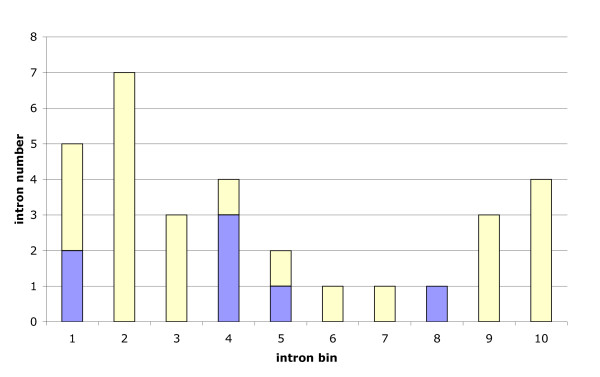
**Relative locations of novel introns supported by NIPs**. Reference for the determination of the intron position within the genes is *D. melanogaster*. The CDS were split into 10 bins of equal size. The numbers of TD introns (blue) and apomorphic ON introns (yellow) were counted. Compared to a uniform distribution both classes together show a 5' bias (p = 0.035; one-sided, binomial test).

To distinguish between the two alternative pathways, we evaluated the splice sites, the proto-splice sites and the relative location of the new introns in more detail:

(1) We determined scores for reference splice sites and proto-splice sites excluding the central AG/GY motif. These scores are based on the nts +3 to +6 (donor site) and the nts -13 to -3 (acceptor site). We found that such partial proto-splice acceptor sites of TD introns show intermediate scores between reference splice sites (RS) on the one hand, and potential proto-splice sites surrounding reference introns (RP) and intron-less AGGY proto-splice site motifs of exons (IS) (Figure 4E) on the other hand. Moreover, the partial proto-splice acceptor site scores of the TD introns are statistically significantly different (Welch t-test, two-sided) from the corresponding scores of other NIP introns (ON) (p = 4.13e-06), reference introns (RP) (p = 1.95e-05) and exonic AGGY motifs (IS) (p = 1.33e-07), respectively. In contrast, the scores of partial proto-splice donor sites show no differences (Figure 4D). This argues against reverse splicing that would predict an exclusive similarity to the donor site. During splicing, the acceptor-binding parts of the spliceosome are occupied by intronic sequences, thus, these parts cannot be involved in target site selection in case of reverse splicing.

(2) If the supposedly duplicated sequences of both intron/exon borders of one intron are compared under exclusion of the central AG/GY motif, these regions of TD introns are significantly more similar to each other compared to other NIP introns (ON) or reference introns (RS/RP) (Welch two-sample t-tests, p = 0.001426 and p = 0.000798, respectively; Figure 4G). This finding is based on the same data as the first one but they were used here in a slightly different way. This result argues in favour of tandem duplication.

(3) The location of the TD introns within the genes does not support an origin by reverse splicing. A reverse-transcription mediated pathway would imply a 3' biased distribution of novel introns as the reverse transcriptase should start primarily at the 3' end of the mRNA [40]. Apomorphic NIP introns, however, show a biased location in favour of the 5' and, to a lesser extent, 3' ends of the genes (Figure 5). Whereas the average locations of apomorphic introns of the TD and ON sets across the 10 bins were not significantly different (p = 0.431; two-sided, Welch two sample t-test), there is a moderate significance (p = 0.035; one-sided, binomial test; 5' -region: bins 1 to 5, 3' -region: bins 6 to 10) to reject the null hypothesis that the 31 novel introns (of both sets ON and TD) are equally distributed between the 5' -region and the 3' -region. This is more consistent with an origin by tandem duplication, which is indifferent to location, than by reverse splicing.

The above suggested cases of exon duplications are not supported by sequence conservation of (surrounding) exonic and unconstrained intronic sequences. This could be due to the relatively large evolutionary age of all cases of exon duplications reported here. The youngest intron that may have originated by duplication has evolved in the *D. melanogaster *lineage after divergence from the *D. erecta *lineage and thus appears to be between 5 and 10 million years old (FBgn0002526, 1460-0). Accordingly, the footprint of duplication might have been lost.

**Figure 6:**
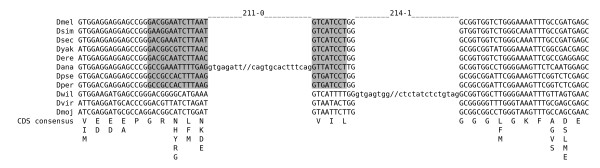
**An example for exon duplication**. Shown is the NIP region of the CG8105 gene (FBgn0030661). Intron sequences are shown in lower case. The amino acid sequence consensus of the CDS is given beneath the exon sequences. While intron 211-0 of *D. ananassae *has probably risen by exon duplication, the mechanism of origination of intron 214-1 (*D. willistoni*) remained unknown. Note that the 5' part of the proto-splice site in position 211-0 (highlighted in grey) strongly varies between the species. No other insect species has an intron within this region of the CDS, so both introns are considered novel.

It has to be noted that intron gain by exon duplication typically cannot be detected using the NIP approach because in most cases an ancient intron will not be present. Thus, tandem duplication may be a more common mechanism of intron gain than suggested by our specifically selected data [19]. However, two different intron positions may arise in one duplicated region if two proto-splice sites are included. In this case, the intron gains are not independent. This may have occurred in FBgn0038300 (introns 44-0 and 54-0) and in FBgn0029747 (introns 200-0 and 207-0) before the divergence of the subgenera *Drosophila *and *Sophophora*. Alternatively, there might have been an ancient intron that was lost before the duplication (FBgn0050101). In the remaining two cases, the other intron seems to be novel, too, but has emerged in another lineage by an unknown mechanism (FBgn0030661 and FBgn0002526, Figure 6).

## Conclusions

**Figure 7:**
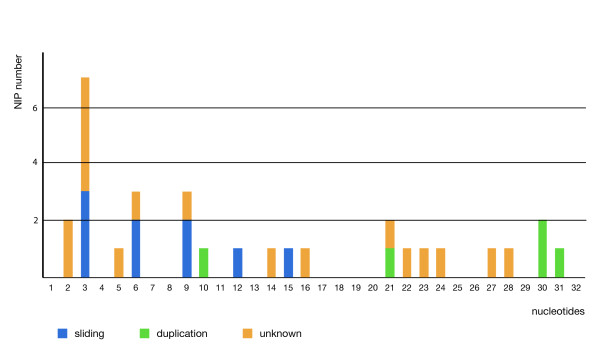
**Distribution of NIP distances**. Distribution of all 31 NIPs according to their intron distance (nts). The supposed evolutionary event for the origin of the novel position is indicated.

During our study of novel intron positions in evolutionarily conserved genes of Drosophila we confirmed that near intron pairs (NIPs) are reliable phylogenetic markers. Furthermore, our results support that intron sliding (migration) is one of the causes of recently emerged intron positions within conserved protein-coding Drosophila genes. We also found evidence for the rise of novel introns by tandem duplication of exonic DNA. The origin for 17 out of 31 identified NIPs remains unknown (Figure 7). Contrary to expectations, the gain of novel introns by other mechanisms could not be proved, for example, by insertion of a spliceosomal intron via reverse splicing into a new position, by insertion of a transposable element or by gene conversion with an intron-containing paralog. Recent origins of spliceosomal introns in eukaryotic genes, therefore, often might be local mutations (Drosophila) or insertions of alien sequences (Daphnia) [14], but not insertions of reverse-transcribed sequences. This is supported by a recent analysis of intron gain through intronization in Caenorhabditis [3] and should be evaluated also for other eukaryotes.

## Methods

### Compilation of the data set

We started with 12386 sets of 1:1 orthologous protein coding genes extracted from FlyBase and the "FlyBase melanogaster gene ortholog report" (release FB2009_01) [41]. For each set, we obtained the available gene sequences (genomic DNA) as well as their translations (protein sequences). We run a NIP extraction alignment procedure as described in more detail in the following, which aims at identifying orthologous and nearby intron positions within the data set.

### Multiple codon alignment and subsequent extraction of NIP region candidates

CDS were compiled according to the FlyBase peptide annotations and aligned at the protein level. The translated multiple alignments (codon alignments) were performed using a slightly modified version of the TransAlign utility [42]. Our modifications included the usage of Muscle [43] for protein alignment and subsequent use of the realignment tool of Csurös et al. [44] to optimize the recognition of conserved intron positions. The other parameters used for the TransAlign utility ensured the exclusion of sequences where stop codons occurred within the CDS.

By mapping the various intron positions onto the multiple translated alignment, orthologous and nearby intron positions become apparent. In cases where several translations (isoforms) for the reference species (*D. melanogaster*) are available, the annotation and reference protein were selected according to highest sequence similarity to all other species' translations. Intron positions within the multiple alignments were named according to their absolute positions within the *Dme *reference peptide sequence by using translated BLAT [45]. In a last step, only those intervals were extracted from each alignment where at least two intron positions were included while separated by less than 50 nt. Regions hereby were extracted including 30 nt flanking alignment sequence around the regions' outmost intron positions.

### Filter steps to remove low confidence and erroneous NIP candidates

We obtained 4044 putative NIP regions each containing at least two intron positions. We excluded NIPs from this set that contained genes with both introns of a pair and such which failed to show the splice rule consensus for both donor (GYR) and acceptor (HAG). In addition, we required at least 6 non-gap characters at both exon borders. This resulted in 959 candidate NIP regions, which we ordered according to a conservation score collected from the most similar pair of amino acid sequences representing different intron positions of the local multiple alignment. The conservation score was calculated as the average of relative sum-of-pair scores utilizing the BLOSUM45 substitution matrix of Clustal W [46]. By requiring a minimal conservation score of 0.75, we reduced the candidate list to 138 potential NIP regions. To enrich the candidates with information on available EST data, we performed an automated search for ESTs overlapping occupied intron positions out of these candidates for each species (NCBI-BLAST-2.2.19+ against NCBI est_others database). Furthermore, we used Drosophila splice site matrices [27] and a weighted score cutoff of 50 to remove sequences and consequently NIPs by detecting obviously falsely annotated intron positions.

The computational filtering concluded with 122 NIP regions that were then inspected manually for validity, resulting in 36 NIPs. During this last step, we also required the valid NIP distance to be less than 32 nt (initially < 50 nt), to obtain a more reliable data set, based on a comparison of the abundances of short exons and NIP distances (data not shown) within Drosophila. The manual analysis included the search for orthologous outgroup sequences to infer the plesiomorphic and apomorphic intron positions where possible. Additionally, 2 Drosophila sequences were manually added to the alignments (e.g. *Dmo *to FBgn0002526), 8 sequences were removed (e.g. *Dgr *from FBgn0015572), and one obvious misannotation/sequence error could be corrected (*Der *in FBgn0046689) (see also comments in Additional file 1).

### PCR analysis

We performed PCR to confirm the intron positions of 7 NIPs that were only weakly supported. For this purpose, we isolated female whole body RNA and genomic DNA from adults of the appropriate Drosophila species. In case of *Drosophila sechellia, D. willistoni *and *D. persimilis*, we used the strains (UC Drosophila Species Stock Center, San Diego, California) that were originally used for whole genome sequencing [18]. The total RNA was subsequently converted to cDNA (primed with Oligo dT) with the RevertAid H Minus First Strand cDNA Synthesis Kit (Fermentas). The primers used for PCR are given in Additional file 3. The resulting PCR products were extracted from agarose gels (Invisorb Spin DNA Extraction Kit, Invitek) and directly sequenced using Big Dye sequencing chemistry (ABI). For sequence analysis, Mac Vector version 7.2 was used.

### Construction of an intron pair distribution matrix

From the 31 remaining NIPs, an intron pair matrix was manually created and analyzed in MacClade 4.0 [47] (Additional file 4). Within the intron pair matrix, the upstream intron is coded as "1", and the downstream intron as "2" or "3" whereas intron less pair positions and no data are coded as "?". Intron data from the insect outgroup species were combined into an "outgroup" row.

### Novel intron sequence analysis

The intron sequences of all 31 final NIPs were compared by BLAST with introns and exons of the corresponding orthologous genes, to identify any similarities. Furthermore, Drosophila and Wolbachia genomic sequences, repetitive sequences [48] and the complete GenBank database (wgs/nr) were used as targets in BLAST analysis. By requiring a nearly complete coverage (max 5 nt unmatched positions at both ends) of the intron query sequence within the target sequences, and a standard E-value cutoff of 0.0001, the BLAST results were filtered on significant hits for probable sources of intron sequences. Additionally, RNAz [49] was used to screen for potentially conserved (thermodynamically stable) secondary structures within each set of aligned (positional orthologous) intron sequences (Clustal W, Muscle).

### Reference data sets and splice site scores

Reference data sets of splice sites were compiled to compare the splice site strengths of potential proto-splice sites. The reference set of in total 16766 splice sites (RS) was created from introns of all transcripts of *D. melanogaster *with a maximal evidence of expression (15 points) [41]. A second reference set of potential proto-splice sites consisting of all exonic sequences immediately surrounding these introns (RP) was created. As a third reference set (IS), all exonic sequences (from the *D. melanogaster *transcripts) were sampled that contain the AGGY proto-splice site motif and whose potential intron positions would be located more than 31 nt from the exon borders. Position weight matrices (PWMs) for splice site scoring were generated from the reference splice sites (RS). For each (proto-) splice site the score was calculated according to [27], i.e. using 3 exonic and 6 intronic positions of the donor site (proto-splice site: 3 positions before and 6 positions after the intron) and 13 intronic and 1 exonic positions of the acceptor site (proto-splice site: 13 positions before and 1 position after the intron). The percentiles of these scores among the reference scores RS and RP were collected (Table 2).

## Abbreviations

CDS: coding sequence; *Dan: Drosophila ananassae; Der: Drosophila erecta; Dgr: Drosophila grimshawi; Dme: Drosophila melanogaster; Dmo: Drosophila mojavensis; Dpe: Drosophila persimilis, Dps: Drosophila pseudoobscura; Dse: Drosophila sechellia; Dsi: Drosophila simulans; Dvi: Drosophila virilis; Dwi: Drosophila willistoni; Dya: Drosophila yakuba*; FBgn: FlyBase identifier number for genes; NIP: near intron pair; nt: nucleotide.

## Authors' contributions

JL carried out the bioinformatic acquisition and data analysis, performed statistical analyses, participated in the design of the study and helped to draft the manuscript. CE performed the PCR analysis and helped to draft the manuscript. PFS participated in the design of the study and helped to draft the manuscript. VK conceived the study, participated in its design and analysis and drafted the manuscript. All authors read and approved the final manuscript.

## Supplementary Material

Additional file 1 **Table of NIP data**. This file contains data about plesiomorphic and apomorphic intron positions, their support by ESTs, intron-less species, RT-PCR, CG number and regional amino acid conservation (PDF format). Click here for file

Additional file 2 **NIP alignments**. Alignments of all Drosophila NIP regions (Text format). Click here for file

Additional file 3 **Table of PCR analysis results of selected NIP candidates**. This file contains the PCR primer sequences and validation results for the selected NIP candidates that were in doubt concerning the reliability of the shifted intron position (PDF format). Click here for file

Additional file 4 **Intron pair matrix**. This file contains the coded intron position data of 31 NIPs. It was used to build the tree of Figure 2 (Nexus format). Click here for file

## References

[B1] Carmel L Wolf YI Rogozin IB Koonin EV Three distinct modes of intron dynamics in the evolution of eukaryotes Genome Res 2007 17 1034 1044 10.1101/gr.6438607 17495008 PMC1899114

[B2] Roy SW Penny D A very high fraction of unique intron positions in the intron-rich diatom Thalassiosira pseudonana indicates widespread intron gain Mol Biol Evol 2007 24 1447 1457 10.1093/molbev/msm048 17350938

[B3] Irimia M Rukov JL Penny D Vinther J Garcia-Fernandez J Roy SW Origin of introns by 'intronization' of exonic sequences Trends Genet 2008 24 378 381 10.1016/j.tig.2008.05.007 18597887

[B4] Roy SW Intronization, de-intronization and intron sliding are rare in Cryptococcus BMC Evol Biol 2009 9 192 10.1186/1471-2148-9-192 19664208 PMC2740785

[B5] Zhu Z Zhang Y Long M Extensive Structural Renovation of Retrogenes in the Evolution of the Populus Genome Plant Physiol 2009 151 1943 1951 10.1104/pp.109.142984 19789289 PMC2785971

[B6] Roy SW Irimia M Mystery of intron gain: new data and new models Trends Genet 2009 25 67 73 10.1016/j.tig.2008.11.004 19070397

[B7] Rogers JH How were introns inserted into nuclear genes? Trends Genet 1989 5 213 216 10.1016/0168-9525(89)90084-X 2551082

[B8] Sharp PA On the origin of RNA splicing and introns Cell 1985 42 397 400 10.1016/0092-8674(85)90092-3 2411416

[B9] Crick F Split genes and RNA splicing Science 1979 204 264 271 10.1126/science.373120 373120

[B10] Hankeln T Friedl H Ebersberger I Martin J Schmidt ER A variable intron distribution in globin genes of Chironomus: evidence for recent intron gain Gene 1997 205 151 160 10.1016/S0378-1119(97)00518-0 9461389

[B11] Gilbert W de Souza SJ Long M Origin of genes Proc Natl Acad Sci USA 1997 94 7698 7703 10.1073/pnas.94.15.7698 9223251 PMC33679

[B12] Giroux MJ Clancy M Baier J Ingham L McCarty D Hannah LC De novo synthesis of an intron by the maize transposable element Dissociation Proc Natl Acad Sci USA 1994 91 12150 12154 10.1073/pnas.91.25.12150 7991598 PMC45394

[B13] Rogozin IB Lyons-Weiler J Koonin EV Intron sliding in conserved gene families Trends Genet 2000 16 430 432 10.1016/S0168-9525(00)02096-5 11050324

[B14] Li W Tucker AE Sung W Thomas WK Lynch M Extensive, recent intron gains in Daphnia populations Science 2009 326 1260 1262 10.1126/science.1179302 19965475 PMC3878872

[B15] Krauss V Thümmler C Georgi F Lehmann J Stadler PF Eisenhardt C Near intron positions are reliable phylogenetic markers: an application to holometabolous insects Mol Biol Evol 2008 25 821 830 10.1093/molbev/msn013 18296416

[B16] Saeys Y Rouzé P Peer Y Van de In search of the small ones: improved prediction of short exons in vertebrates, plants, fungi and protists Bioinformatics 2007 23 414 420 10.1093/bioinformatics/btl639 17204465

[B17] Weir M Eaton M Rice M Challenging the spliceosome machine Genome Biol 2006 7 3 10.1186/gb-2006-7-1-r3 PMC1431713 16507135

[B18] Drosophila 12 Genomes Consortium Evolution of genes and genomes on the Drosophila phylogeny Nature 450 203 218 10.1038/nature06341 17994087

[B19] Coulombe-Huntington J Majewski J Intron loss and gain in Drosophila Mol Biol Evol 2007 24 2842 2850 10.1093/molbev/msm235 17965454

[B20] Farlow A Meduri E Dolezal M Hua L Schlötterer C Nonsense-mediated decay enables intron gain in Drosophila PLoS Genet 2010 6 e1000819 10.1371/journal.pgen.1000819 20107520 PMC2809761

[B21] Gilbert DG DroSpeGe: rapid access database for new Drosophila species genomes Nucleic Acids Res 2007 35 D480 485 10.1093/nar/gkl997 17202166 PMC1899099

[B22] Hiller M Nikolajewa S Huse K Szafranski K Rosenstiel P Schuster S Backofen R Platzer M TassDB: a database of alternative tandem splice sites Nucleic Acids Res 2007 35 D188 192 10.1093/nar/gkl762 17142241 PMC1669710

[B23] Horowitz DS Krainer AR Mechanisms for selecting 5' splice sites in mammalian pre-mRNA splicing Trends Genet 1994 10 100 106 10.1016/0168-9525(94)90233-X 8178363

[B24] Roca X Sachidanandam R Krainer AR Determinants of the inherent strength of human 5' splice sites RNA 2005 11 683 698 10.1261/rna.2040605 15840817 PMC1370755

[B25] Buratti E Chivers M Královicová J Romano M Baralle M Krainer AR Vorechovský I Aberrant 5' splice sites in human disease genes: mutation pattern, nucleotide structure and comparison of computational tools that predict their utilization Nucleic Acids Res 2007 35 4250 4263 10.1093/nar/gkm402 17576681 PMC1934990

[B26] Vorechovský I Aberrant 3' splice sites in human disease genes: mutation pattern, nucleotide structure and comparison of computational tools that predict their utilization Nucleic Acids Res 2006 34 4630 4641 10.1093/nar/gkl535 16963498 PMC1636351

[B27] Sheth N Roca X Hastings ML Roeder T Krainer AR Sachidanandam R Comprehensive splice-site analysis using comparative genomics Nucleic Acids Res 2006 34 3955 3967 10.1093/nar/gkl556 16914448 PMC1557818

[B28] Sverdlov AV Rogozin IB Babenko VN Koonin EV Evidence of splice signal migration from exon to intron during intron evolution Curr Biol 2003 13 2170 2174 10.1016/j.cub.2003.12.003 14680632

[B29] Lynch M Richardson AO The evolution of spliceosomal introns Curr Opin Genet Dev 2002 12 701 710 10.1016/S0959-437X(02)00360-X 12433585

[B30] Lim LP Burge CB A computational analysis of sequence features involved in recognition of short introns Proc Natl Acad Sci USA 2001 98 11193 11198 10.1073/pnas.201407298 11572975 PMC58706

[B31] Jaillon O Bouhouche K Gout JF Aury JM Noel B Saudemont B Nowacki M Serrano V Porcel BM Ségurens B Le Mouël A Lepère G Schächter V Bétermier M Cohen J Wincker P Sperling L Duret L Meyer E Translational control of intron splicing in eukaryotes Nature 2008 451 359 362 10.1038/nature06495 18202663

[B32] Dibb NJ Newman AJ Evidence that introns arose at proto-splice sites EMBO J 1989 8 2015 2021 2792080 10.1002/j.1460-2075.1989.tb03609.x PMC401080

[B33] Venkatesh B Ning Y Brenner S Late changes in spliceosomal introns define clades in vertebrate evolution Proc Natl Acad Sci USA 1999 96 10267 10271 10.1073/pnas.96.18.10267 10468597 PMC17877

[B34] Zhuo D Madden R Elela SA Chabot B Modern origin of numerous alternatively spliced human introns from tandem arrays Proc Natl Acad Sci USA 2007 104 882 886 10.1073/pnas.0604777104 17210920 PMC1783408

[B35] Roy SW Irimia M When good transcripts go bad: artifactual RT-PCR 'splicing' and genome analysis Bioessays 2008 30 601 605 10.1002/bies.20749 18478540

[B36] Sverdlov AV Rogozin IB Babenko VN Koonin EV Reconstruction of ancestral protosplice sites Curr Biol 2004 14 1505 1508 10.1016/j.cub.2004.08.027 15324669

[B37] Emerson JJ Cardoso-Moreira M Borevitz JO Long M Natural selection shapes genome-wide patterns of copy-number polymorphism in Drosophila melanogaster Science 2008 320 1629 1631 10.1126/science.1158078 18535209

[B38] Kershaw CJ Barrass JD Beggs JD O'Keefe RT Mutations in the U5 snRNA result in altered splicing of subsets of pre-mRNAs and reduced stability of Prp8 RNA 2009 15 1292 1304 10.1261/rna.1347409 19447917 PMC2704078

[B39] Crotti LB Bacíková D Horowitz DS The Prp18 protein stabilizes the interaction of both exons with the U5 snRNA during the second step of pre-mRNA splicing Genes Dev 2007 21 1204 1216 10.1101/gad.1538207 17504938 PMC1865492

[B40] Sverdlov AV Babenko VN Rogozin IB Koonin EV Preferential loss and gain of introns in 3' portions of genes suggests a reverse-transcription mechanism of intron insertion Gene 2004 338 85 91 10.1016/j.gene.2004.05.027 15302409

[B41] FlyBase A Database of Drosophila Genes & Genomes http://flybase.org

[B42] Bininda-Emonds ORP transAlign: using amino acids to facilitate the multiple alignment of protein-coding DNA sequences BMC Bioinformatics 2005 6 156 10.1186/1471-2105-6-156 15969769 PMC1175081

[B43] Edgar RC MUSCLE: multiple sequence alignment with high accuracy and high throughput Nucleic Acids Res 2004 32 1792 1797 10.1093/nar/gkh340 15034147 PMC390337

[B44] Csurös M Holey JA Rogozin IB In search of lost introns Bioinformatics 2007 23 i87 i96 10.1093/bioinformatics/btm190 17646350

[B45] Kent WJ BLAT - the BLAST-like alignment tool Genome Res 2002 12 656 664 11932250 10.1101/gr.229202 PMC187518

[B46] Larkin MA Blackshields G Brown NP Chenna R McGettigan PA McWilliam H Valentin F Wallace IM Wilm A Lopez R Thompson JD Gibson TJ Higgins DG Clustal W and Clustal X version 2.0 Bioinformatics 2007 23 2947 8 10.1093/bioinformatics/btm404 17846036

[B47] Maddison DR Maddison WP MacClade 4.08 2005 Sunderland (MA): Sinauer Associates

[B48] Repbase GIRI http://www.girinst.org/repbase/index.html

[B49] Washietl S Prediction of structural noncoding RNAs with RNAz Methods Mol Biol 2007 395 503 526 17993695 10.1007/978-1-59745-514-5_32

