# Supplementary material for: Some novel intron positions in conserved Drosophila genes are caused by intron sliding or tandem duplication
Source: BMC Evol Biol. 2010 May 26;10:156. doi: 10.1186/1471-2148-10-156 (PMC2891723; doi:10.1186/1471-2148-10-156)
Supplement: Additional file 1 — Table of NIP data. This file contains data about plesiomorphic and apomorphic intron positions, their support by ESTs, intron-less species, RT-PCR, CG number and regional amino acid conservation (PDF format). [file 1471-2148-10-156-S1.PDF]

Additional file 1 - Table of NIP data

nt	FBgn (FlyBase gene ID)	Plesiomorphic intron (confirmed by ESTs if bold)	Apomorphic intron (confirmed by ESTs if bold)	Intron-less	Apomorphic subtree node	RT-PCR	origin	Gene name conservation score
2	0031216		1927-1: vir, moj, gri<br>1928-0: mel, sim, sec, yak, ere, ana, per, pse, wil	Aga, Cpi, Aae			Unknown	CG11376<br>0.932
2	0036142		456-2: vir, moj, gri<br>456-0: wil, per, pse, ana, ere, yak, sec, sim, mel	Aga, Cpi, Phu, Gmo (457-2)			Putative sliding	CG7616<br>0.955
3	003607	117-1: **gri**, vir, moj, **wil**, **ana**	118-1: **mel**, **sim**, sec, **yak**, ere, pse, per		(5, 9)		Sliding	Su(var)2-5, CG8409<br>0.887
3	0015572	5-2: **mel**, sim, ere, yak, ana, wil, moj, vir	4-2: per, pse		9	pse, per	Putative sliding	Alpha-esterase 4, CG1082<br>0.779
3	0032087	20-1: mel, sec, sim, yak,ere, ana, per, pse, wil, moj, vir	19-1: **gri**		D.grimshawi		Putative sliding	CG9568<br>0.783
3	0032261	56-0: wil, gri, vir, moj	55-0: **ana**, per, pse, (**mel**, sim, sec, yak, ere)		3		Sliding	CG6094<br>0.916
3	0032504	198-2: mel, sim, ere<br>199-2: pse, per		ana, wil, vir			Unknown	CG16970<br>1.000
3	0037757	615-1: per, pse, wil, moj, vir, gri	616-1: mel, sec, ere, yak, ana		4		Sliding	CG8516<br>0.843
3	0052081	4-2: ana<br>5-2: mel, sim, yak, ere		(wil, vir, moj excluded from alignment)			Putative sliding	CG32081<br>0.868

Additional file 1 - Table of NIP data

nt	FBgn (FlyBase gene ID)	Plesiomorphic intron (confirmed by ESTs if bold)	Apomorphic intron (confirmed by ESTs if bold)	Intron-less	Apomorphic subtree node	RT-PCR	origin	Gene name conservation score
5	0046689	15-0: **mel**, sim, sec, per, pse, yak, ere	16-2: ana	(ere annotation manually improved)	D.ananassae	ana	Putative sliding	Tak1-like 1, CG31421 0.752
6	0019809	253-0: moj, vir, gri, wil, sec, sim, **mel**, yak, ere, ana	255-0: pse, per		9		Sliding	gcm2, CG3858 0.958
6	0031395	40-0: mel, sim, sec, yak, ere, ana, pse, per, wil<br>42-0: gri, moj, vir					Putative sliding	CG10874 0.891
6	0035879	103-1: **pse**, per, ere, yak, sim, sec, mel	105-1: ana	(wil vir moj gri excluded from alignment)			Sliding	CG7112 0.851
9	0032821	1197-0: **mel**, sim, sec, yak, ere, ana, pse, per, wil, **vir**, gri	1200-0: moj		D.mojavensis		Sliding	CdGAPr, CG10538 0.814
9	0034221	396-1: yak<br>399-1: mel, sim sec					Sliding	CG10764 0.809
9	0038302	68-2: mel, sim, sec, yak, ere, ana, per, pse, gri, moj, vir	65-2: wil		D.willistoni		Putative sliding	CG4210 0.783
10	0030661	211-0: ana<br>214-1: wil		mel, sim, sec, yak, ere, pse, per, vir, moj			Exon duplication	CG8105 0.852

Additional file 1 - Table of NIP data

nt	FBgn (FlyBase gene ID)	Plesiomorphic intron (confirmed by ESTs if bold)	Apomorphic intron (confirmed by ESTs if bold)	Intron-less	Apomorphic subtree node	RT-PCR	origin	Gene name conservation score
12	0033734	420-0: ana, pse, per, **wil**, Phu, Bmo	419-0: mel, sim, sec, yak, ere<br>423-0: vir, moj, gri	Aga, Aae, Cpi	5<br>10		Sliding	CG8520<br>0.897
14	0035965	48-2: **gri**, **moj**, vir, Aga, Aae, Cpi, Gmo, Phu, Bmo	44-0: wil	mel, sim sec, yak, ere, ana, per, pse	D.willistoni		Unknown	Use1, CG14181<br>0.866
15	0001124	119-0: **sec**, **sim**, **mel**, ere, yak, ana, per, **pse**, gri, vir, moj, Bmo, Phu, Tca, Gmo	124-0: **wil**		D.willistoni		Sliding	CG8430, Got1<br>1.000
16	0033686		53-2: gri, moj, vir<br>59-0: wil	mel, sim, sec, yak, ere, ana, pse, per			Unknown	piRNA methyltransferase, CG12367<br>0.836
21	0029747	200-0: mel, sim, sec, yak, ere, wil<br>207-0: vir, moj, (gri)		pse, per, ana			Exon duplication	CG5062<br>0.810
21	0030055	87-0: mel, sim, ere, sec, yak, ana, wil (pse,per)<br>94-0: gri, moj, vir		(manually aligned)			Putative sliding	CG12772<br>0.770
22	0032517	213-2: sec, sim, mel, ere, yak, ana, pse, moj, vir , gri	221-0: wil		D.willistoni		Unknown	CG7099<br>0.832

Additional file 1 - Table of NIP data

nt	FBgn (FlyBase gene ID)	Plesiomorphic intron (confirmed by ESTs if bold)	Apomorphic intron (confirmed by ESTs if bold)	Intron-less	Apomorphic subtree node	RT-PCR	origin	Gene name conservation score
23	0033247	38-0: sec, sim, **mel**, ere, yak, **ana**, **pse**, per moj, vir, **gri**, Isc, Gmo, Bmo, Aga, Aae, Cpi, Phu, Tca	45-2: **wil**		D.willistoni		Unknown	CG8722, Nup44A<br>0.949
24	0034793	21-0: **sec**, **mel**, ere, yak, ana, pse, per, **moj**, vir, **gri**	29-0: **wil**		D.willistoni		Putative sliding	asrij, CG13533<br>0.870
27	0001185	44-2: wil<br>53-2: ana		mel, sim, sec, yak, ere, pse, per, vir, moj, gri			Unknown	hermaphrodite, CG4694<br>0.775
28	0031773	155-0: **mel**, sim, sec, yak, ere, ana, pse, per, wil, Aae, Aga, Cpi, Tca	164-1: vir, moj, gri		10		Unknown	CG9144<br>1.000
30	0038300	44-0: wil<br>54-0: gri, moj, vir		mel, (sim), sec, yak, ere, ana, pse, per, Isc, Aga, Aae, Gmo, Bmo, Phu, Tca			Exon duplication	CG4203<br>0.979
30	0050101	241-0: sec, sim, **mel**, ere, yak, ana, pse, per, moj, vir, gri	251-0: wil		D.willistoni		Exon duplication	CG30101<br>0.993

Additional file 1 - Table of NIP data

nt	FBgn (FlyBase gene ID)	Plesiomorphic intron (confirmed by ESTs if bold)	Apomorphic intron (confirmed by ESTs if bold)	Intron-less	Apomorphic subtree node	RT-PCR	origin	Gene name conservation score
31	0002526	1460-0: mel, sim, sec, yak<br>1449-2: gri, moj, vir		ere, ana, pse, per, wil, Api, Tca, Aga, Cpi, Nvi, Ame, Aae, Bmo			Exon duplication	Laminin A, CG10236<br>0.940

Intron positions with species names in bold letters indicate that these positions are supported by EST hits.
Abbreviations: mel=Drosophila melanogaster, sim=D. simulans, sec=D. sechellia, yak=D. yakuba, ere=D. erecta, ana= D. ananassae, pse= D. pseudoobscura, per=D. persimilis, wil= D. willistoni, vir=D. virilis, moj=D. mojavensis, gri=D. grimshawi, Aae=Aedes aegypti, Aga=Anopheles gambiae, Api=Acyrthosiphon pisum, Cpi=Culex pipiens, Bmo=Bombyx mori, Tca=Tribolium castaneum, Ame=Apis mellifera, Nvi=Nasonia vitripennis, Phu=Pediculus humanus, Gmo=Glossina morsitans
