# Supplementary material for: Some novel intron positions in conserved Drosophila genes are caused by intron sliding or tandem duplication
Source: BMC Evol Biol. 2010 May 26;10:156. doi: 10.1186/1471-2148-10-156 (PMC2891723; doi:10.1186/1471-2148-10-156)
Supplement: Additional file 3 — Table of PCR analysis results of selected NIP candidates. This file contains the PCR primer sequences and validation results for the selected NIP candidates that were in doubt concerning the reliability of the shifted intron position (PDF format). [file 1471-2148-10-156-S3.PDF]

Additional file 3 - Table of PCR analysis results of selected NIP candidates "nt" means NIP distance in nucleotides.

nt	FBgn	Plesiomorphic intron	Supposed apo-morphic intron	NIP confirmed	Evidence	Used primers
1	36324	155-1: Dme, Dsi, Dya, Dan, Dmo, Dgr	155-0: Dse	no	Genomic PCR (Dse)	Dse: 5'-CACCGATTCAAAAGCCATTC-3', 5'- TTTCACCAGAGTTGCGAGTG-3'
3	15572	5-2: Dme, Dsi, Dya, Der, Dan, Dwi, Dmo, Dvi	4-2: Dpe, Dps	yes	RT-PCR (Dpe, Dps)	Dps, Dpe: 5'-TTTCACCAGAGTTGCGAGTG-3', 5'- CCATGCCAGGGCTTCAG -3'
4	27055	314-0: Dme, Dse, Dya, Der, Dan, Dps, Dpe, Dwi	315-1: Dsi	no	RT-PCR (Dsi, Dme, Dse, Dya, Der, Dps, Dpe)	Dps, Dpe: 5'-AGCGTTTATACCAAAAAACACAC-3', 5'- TGTTCAGGATGTAGCGTTCTG-3'; Dya, Der: 5'-CCGTTTTATGAAGCCGATG-3', 5'-CCATGTTGAGAATGTAACGCTC-3'; Dme, Dsi, Dse: 5'-CCAAAAAACACACAGGTCATTG-3', 5'-CCATGTTGAGAATGTAACGCTC-3'
4	38858	116-0: Dme, Dsi, Dse, Dya, Der, Dan, Dpe, Dps, Tca	117-1: Dwi	no	RT-PCR (Dwi)	Dwi: 5'-CCGACCACATCGTATCTGC-3', 5'-TTCTGTTTCACCTCTCCGTTG-3'
5	46689	15-0: Dme, Dsi, Dse, Dya, Dpe, Dps	13-1: Der	no	RT-PCR (Dya, Der)	Dya, Der: 5'-CGCACGTACTGCTAAAAATGG-3', 5'- GCTCCACGGTGTACTCCCAC -3'
5			16-2: Dan	yes	RT-PCR (Dan)	Dan: 5'-CGCAAGATGACCGAAATAGTG-3', 5'-TTTTTGTGCTTCAGTCGTGC-3'
6	82831	750-0: Dme, Dse, Dya, Dan	752-0: Der	no	RT-PCR (Dsi, Dya, Der)	Dme, Dsi, Dse: 5'-GCTGATTTTCTCCAGCACG-3', 5'- TCAATGGGAGGCAGGTATATG-3'; Dya, Der: 5- GCTGTGGCAGATCCGAATG-3', 5'- TGTGCTCGTCGCTTGGC-3'
